# Unexpected custodial death due to acute epiglottitis: A rare autopsy case report

**DOI:** 10.1097/MD.0000000000009941

**Published:** 2018-02-16

**Authors:** Shifan Wu, Luo Zhuo, Xingang Qiu, Zijiao Ding, Mingzhen Yang, Meichen Pan, Qian Liu

**Affiliations:** a Department of Forensic Medicine, Tongji Medical College of Huazhong University of Science and Technology, Wuhan; b Division of Forensic Science, Fujian Provincial Department of Public Security, Fuzhou; c Shanghai Key Laboratory of Forensic Medicine, Shanghai Forensic Service Platform, Institute of Forensic Science, Ministry of Justice, PRC, Shanghai, P.R. China.

**Keywords:** acute epiglottitis, custodial death, forensic pathology, medicine, sudden death

## Abstract

**Rationale:**

Acute epiglottitis is a potentially life-threaten disease, which makes it more challenging to save the life for doctors. Unexpected deaths in custody are a primary cause of concern for the forensic community and doctor worldwide.

**Patient concerns:**

We present a case of a 44-year-old male detainee who was clinically suspected of dying of acute epiglottitis. The man experienced failure of resuscitation and died after admitted to a hospital.

**Diagnoses:**

The autopsy, toxicological testing, the test of immunoglobulin E and bacterial culture suggested the patient died of acute epiglottitis.

**Interventions:**

The bacterial culture was performed to imprecisely identify the cause of death.

**Outcomes:**

The bacterial culture of the patient's heart blood and nasal and throat swabs showed the presence of the pathogenic microorganism Haemophilus influenza type B.

**Lessons:**

We aim to provide a reference to the medical and forensic community and remind the local law enforcement agencies on the problems present within the correctional healthcare system through this case report. Additionally, we also aim to increase the current knowledge and understanding on custodial deaths caused by natural diseases.

## 1 Introduction

Custodial death refers to the death of an individual who is in prison or while under custody,^[1]^ which can be attributed to natural diseases or causes, violence, poisoning, or even accidents.^[2,3]^ Unfortunately, at present, the statistical data on custodial deaths in China are limited. Here, we present the case of a prisoner who died unexpectedly, although he appeared to be healthy while in custody. The result of the postmortem examination determined acute epiglottitis as the cause of death. Custodial deaths due to diseases may expose the limitations of the current health care correctional system, and we aim to arouse the attention of the law enforcement agencies to these limitations through the findings of this case report. Medical Ethics Committee of Tongji Medical College of Huazhong University of Science and Technology approved the research. Written informed consent was obtained by the relatives of the deceased for publication of this report and accompanying images.

## 2 Case report

A 44-year-old male who was taken to a detention center for possession of illegal drugs 6 months ago complained of dyspnea and swelling of the neck. Subsequently, he was admitted to a hospital 4 hours later in a state of deep coma. Before hospital admission, he did not receive any treatment for his symptoms. His temperature was 39.2°C. A computed tomography (CT) scan revealed the narrowing of the pharynx, larynx, and glottis along with the swelling of the right submandibular gland and pharyngeal soft tissue. Resuscitation attempts failed, and the patient soon died after admission with a clinical diagnosis of laryngeal obstruction. On the basis of the medical records, the patient had no history of allergies and trauma to the neck, and he had been healthy throughout his detainment. Further investigation showed that the patient consumed regular diet and did not take any prescribed medications while in the jail. The case was reported to the prosecutor's office, and a forensic autopsy was conducted to identify the cause of the patient's death.

**Figure 1:**
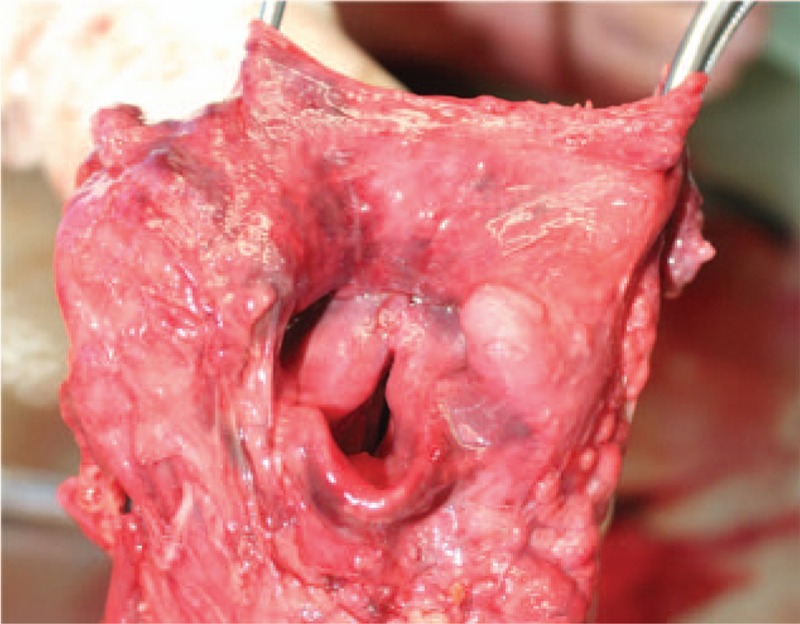
Diffuse swelling of the epiglottis and aryepiglottic folds with redness.

**Figure 2:**
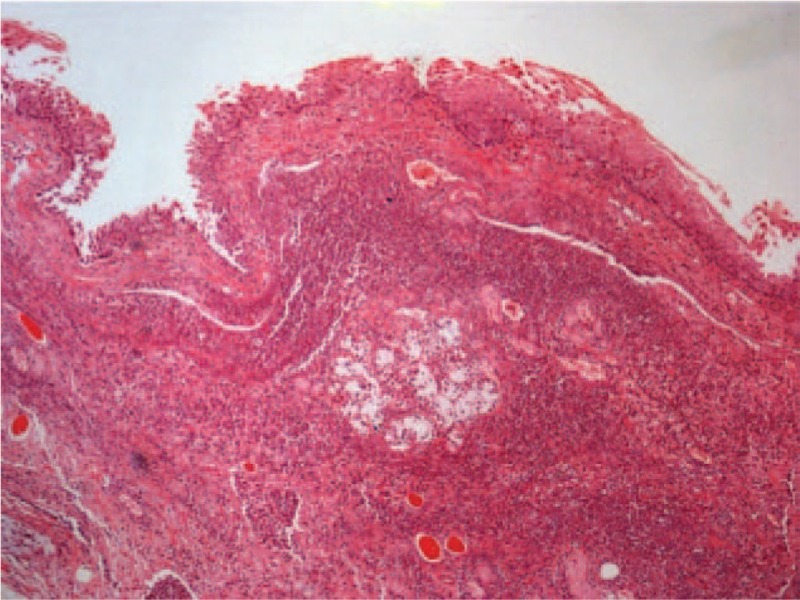
Microscopic finding of the right epiglottitis with hemorrhage, dense inflammatory cell infiltration, and necrotic debris in the mucosal and submucosal tissues (HE, 40×).

The autopsy findings indicated that the patient's body height and weight were 165 cm and 68 kg, respectively. The results of the external examination showed a well-nourished and normally developed body with cyanotic lips and nails. Furthermore, no external injury was found. Meanwhile, the findings of the internal examination revealed normally sized and appropriately placed organs. The neck dissection displayed a swollen epiglottis and aryepiglottic folds characterized by dark red color and thickened margin (Fig. 1). The left and right lungs were significantly edematous and congested, which weighed 830 and 900 g, respectively. Meanwhile, the heart weighed 300 g, with no evidence of coronary atherosclerosis or myocarditis. Under the microscope, the larynx was notably edematous, which was associated with the presence of necrotic debris, extensive hemorrhage, and inflammatory cell infiltration that was restricted to the mucosa and submucosa of the epiglottis (Fig. 2), aryepiglottic folds, and glottis. The assessment of the trachea, bronchi, and lung parenchyma revealed unremarkable findings. The patient's serum immunoglobulin E (lgE) level was within the normal range. The bacterial culture of the patient's heart blood and nose and throat swabs showed the presence of the pathogenic microorganism *Haemophilus influenza* type B.

The samples of the patient's heart blood, urine, stomach contents, and liver tissues were submitted for toxicological testing. A general toxicological screening for ethanol, pesticides, raticides, cyanides, and sedative–hypnotic drugs, illicit drugs, and anesthetics conducted using gas chromatography with mass spectrometry, enzyme immunoassay, and high-performance liquid chromatography revealed negative findings.

## 3 Discussion

Acute epiglottitis is an infection caused by *H. influenzae* and other bacteria as well as fungi and virus.^[4–10]^ This disease is known to be potentially life-threatening because the special structure and anatomical location of the epiglottis make it prone to severe edema, which can lead to dyspnea and even suffocation, resulting in death.^[4,11–13]^ The clinical features of acute epiglottitis commonly include sore throat, odynophagia, stridor, fever, hoarseness, drooling, hyperpyrexia, and dyspnea.^[5,9,10,13–17]^ The typical histopathological changes associated with this condition include extensive mucosal or submucosal edema accompanied by neutrophil infiltration and hemorrhage, which are frequently found microscopically. In addition, epiglottic abscess may be observed in some patients with acute epiglottitis (approximately 24% of the cases).^[11,16,18]^ In this case, the patient experienced shortness of breath, and this condition rapidly progressed to unexpected death. Therefore, the medical staff members should be aware of severe complications related to adult acute epiglottitis and treat the patients immediately once the symptoms appear, especially with regard to the relief of airway obstruction and suffocation.

Aside from the case reported here, other cases of deaths in the correctional system have also been recorded. Therefore, forensic pathologists should also focus on uncovering whether violence or suicide attempts have occurred in the facility. In the case of the patient studied here, the body did not appear to have any evidence of lethal antemortem injuries. Therefore, suicide attempt had been ruled out. The toxicological tests also revealed negative results for the presence of common poisons. Moreover, the negative eosinophil findings and regular serum IgE results eliminated the possibility of allergy as the cause of the patient's death. The CT scan results revealed narrowing of the pharynx, larynx, and glottis. The postmortem examination of the patient also demonstrated severe edema of the epiglottis, which led to fatal airway obstruction. The histology results showed notable edema associated with necrotic debris, extensive hemorrhage, and inflammatory cell infiltration. The postmortem bacterial culture of the patient's heart blood and nose and throat swabs revealed the presence of *H*. *influenza* type B. Therefore, on the basis of the findings, the patient died of acute epiglottitis (caused by *H*. *influenza* type b), and the manner of death was natural.

Currently, the literature on custodial deaths in China is limited. Yang and Li^[19]^ found that the main causes of custodial deaths in China include injuries, suffocation, poisoning, electric shock, and natural diseases. Violent death is the most common cause of custodial deaths, followed by suicide and natural diseases.^[19]^ The health and safety of detainees are both a personal issue and social problem. Furthermore, the occurrence of custodial deaths is closely related not only to human right awareness and national economic development but also to economic and health care conditions at the supervision site. Studies have shown that 36% of custodial deaths were not caused by direct injury but related to postinjury complications. Some Chinese detention centers manage acute diseases based on the terms and conditions and levels of restrictions imposed by these facilities. However, frequent delays in the timing of treatment can lead to controversial deaths. In this case, the detention facility where the patient died lacked vital medical equipment, such as a CT scanner and medical ventilator, which can be detrimental to the diagnosis and treatment of the patient at some level. Therefore, the Chinese government should improve the health care environment within these detention centers.^[19–21]^

We present this case report as a reminder to the medical and forensic community to provide close attention on common but life-threatening diseases that may easily be ignored under some special circumstances. In addition, considering that unexpected death caused by acute epiglottitis is rarely reported in the forensic field, a complete autopsy and pathological and microbiological examinations should be conducted.
